# Hemophilia presenting as recurrent ocular hemorrhage

**DOI:** 10.3205/oc000142

**Published:** 2020-03-18

**Authors:** Luis Miguel Aquino, Felice Katrina Ranche

**Affiliations:** 1 Department of Ophthalmology and Visual Sciences, Philippine General Hospital, Manila, Philippines

**Keywords:** hemophilia, ocular hemorrhage, surgery, bleeding

## Abstract

**Objective:** Patients with hereditary bleeding disorders rarely present with intraocular or orbital hemorrhage as the initial symptom. The presence of such a condition can be easily overlooked when contemplating ophthalmic surgery, and can give rise to intraoperative and postoperative complications. Awareness of such conditions can improve surgical decisions.

**Methods:** This is a case report of an eight-year-old Filipino male who sustained blunt trauma to his right eye, causing traumatic total hyphema with corneal staining. Subretinal hemorrhage was seen on ultrasound. The patient underwent anterior chamber washout with temporary keratoprosthesis and pars plana vitrectomy with silicone oil tamponade. Clearance of the hyphema was noted postoperatively. However, on follow-up after 19 days, the patient presented with recurrence of hyphema, new onset proptosis and peribulbar hemorrhage.

**Results:** Imaging of the orbit revealed new-onset pseudoproptosis with intraocular and peribulbar hemorrhage. A bleeding disorder was suspected at this point. Further probing revealed a family history of prolonged bleeding time in an X-linked genetic inheritance pattern spanning three generations. Laboratory testing of prothrombin, partial thromboplastin, and factor assays were done, which revealed factor VIII deficiency, diagnostic of hemophilia A. No further surgery was done. The patient was given transfusions of fresh frozen plasma, which resolved the hemorrhage.

**Conclusions:** Bleeding disorders present a dilemma in the surgical management of patients. In cases of traumatic hemorrhage, adequate history and physical examination should always be done to rule these out. Surgical outcomes in hemophiliacs can be improved with preoperative prophylactic treatment and close postoperative monitoring and care.

## Introduction

Hemophilia is the most common and serious inherited coagulation factor deficiency. It occurs in 1:5–10000 males in an X-linked inheritance pattern with no racial predilection. Multiple subtypes of hemophilias exist, 85% of which are of the factor VIII deficiency or hemophilia A type. Clinically, however, hemophilias are all virtually identical and manifest in the same way, which is delayed clotting or prolonged bleeding in an area of vascular disturbance [1].

The clinical similarity of all hemophilias is due to the similarity in their pathophysiologies, affecting the same coagulation pathway of the human body, albeit at different points for each type [1]. In the coagulation pathway, thrombin and fibrin formation are the final endpoints required for effective clotting. Each step requires a different factor in order to complete their activation. A deficiency of a factor in a certain step identifies the type of hemophilia present, and prevents completion of the entire pathway.

This then manifests as prolonged bleeding, with the hallmark clinical manifestation of hemophilias being hemarthroses, or bleeding from the joints. Intramuscular bleeding is also common, specifically the iliopsoas. Other bleeds, which include ocular bleeding and trauma, comprise only 5% of reported cases of initial presentation of hemophilia [2].

Ocular hemorrhage can be from both traumatic and non-traumatic causes. In a 2014 meta-analysis of previously published reports, non-traumatic orbital hemorrhage was attributed to inherent bleeding disorders in only 24/124 or 19% of reported cases in literature over the past 30 years. Of these 24 cases, only 1/24 or 0.8% of the total was due to hemophilia, showing the rarity of non-traumatic ocular bleeding as a presentation of hemophilia [3]. Traumatic orbital hemorrhage is more common. However, bleeding from ocular trauma as the only initial manifestation of an underlying hemophilia condition is rare. Hemophiliac patients usually would have presented with other forms of previous bleeding episodes prior [4].

As most of these patients undergo surgery or intervention with the hemophiliac condition usually overlooked/undiagnosed due to incomplete pre-op workup, complications can arise [5]. Some cases of ocular bleeding are also usually dismissed easily or treated routinely, however such presentations in a hemophiliac patient can have underlying serious implications [6]. Paying attention to a visual acuity decrease that is disproportionate to the amount/manner of trauma should therefore alert the attending ophthalmologist to the presence of a more serious underlying condition.

Bleeding into the orbit occurs within a poorly expandable space, causing the globe to proptose anteriorly, acting as a plug to seal off the bleeding. In a hemophiliac patient, continuous bleeding therefore might be occurring, but is not seen on gross exam [7]. Subconjunctival hemorrhage is another clinical finding also commonly signed off with just cold compress as management, but if present in a hemophiliac could actually develop into total peribulbar hemorrhage within 24 hours [6]. All of these cause a decrease in vision due to compressive optic neuropathy, nerve sheath hemorrhage, retinal vein/artery occlusion, or increased intraocular pressure possibly ongoing in a patient but not adequately checked. If left untreated or undiagnosed, therefore, this condition may cause not only permanent damage to the eye, but life-threatening hypovolemic shock, especially if damage to the integrity of the vasculature is severe, such as in trauma, or if there will be surgical manipulation [5].

Hemophilia is an important condition to consider or rule out in patients presenting with any form of unexpected bleeding. Ocular or orbital hemorrhage, while quite common and easily dismissed, can actually be a sign of an ongoing, more emergent condition, hence should never be taken lightly. Diagnosis of hemophilia and physician attention is more vital in these patients who are to undergo any form of surgery, ocular or otherwise, especially children.

## Case description

This was particularly evident in a case of an 8-year-old Filipino male, previously well, who sustained blunt trauma from a small stone thrown from around 10 ft away to his face. The patient was initially noted to have eye redness, pain and decrease in vision. He was managed at a local hospital with topical medications, then sent home and advised further consultation at a tertiary hospital. He was first seen at our institution 10 days post-trauma.

**Figure 1:**
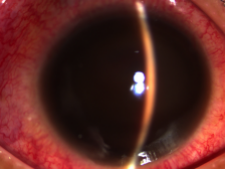
Slitlamp examination of the right eye on initial consult

At this time, the right eye had a visual acuity of light perception, with noted periorbital swelling, erythema of the conjunctivae, and a total hyphema with corneal staining, as seen in Figure 1 (Fig. 1). No gross proptosis or chemosis was noted. Intraocular pressure was 10 mmHg, extraocular muscle movement was full at this time, and the left eye was unremarkable but with a reverse relative afferent pupillary defect. Because of the total hyphema obscuring the posterior pole, ocular ultrasound was done which revealed lens dislocation and a subretinal hemorrhage.

The patient was then assessed as a case of periorbital hematoma, traumatic hyphema with corneal staining of the right eye, with lens dislocation and subretinal hemorrhage by ultrasound. Surgery was then scheduled for the patient, and was cleared by Pediatrics and Anesthesia for surgery, with just the standard laboratory tests of complete blood count, chest radiograph and urinalysis. Penetrating keratoplasty, pars plana vitrectomy, lens extraction, retinotomy, air-fluid exchange and silicone oil injection under general anesthesia were done on the patient. 

**Figure 2:**
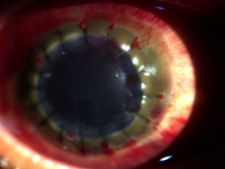
Postoperative day 3 slitlamp examination of the right eye

Postoperative day 1 was uneventful, with no note of refill of the hyphema or increased intraocular pressure. Visual acuity at this time was hand movement with good light projection. However, on the 3^rd^ day, the patient developed fresh blood in the anterior chamber. Figure 2 (Fig. 2) shows the corneal graft was still clear, but there was now increased intraocular pressure. The patient was still sent home on topical antibiotics-steroid, oral acetazolamide and tranexamic acid.

**Figure 3:**
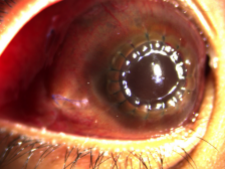
Third-week postoperative slitlamp examination of the right eye

**Figure 4:**
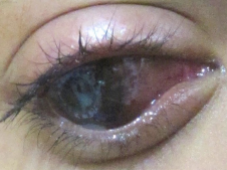
Third-week postoperative gross examination of the right eye

**Figure 5:**
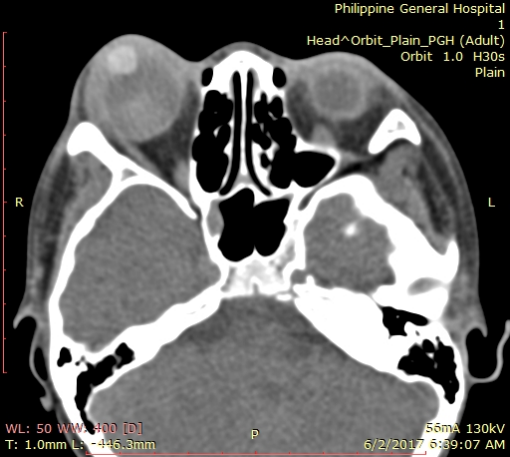
Third-week postoperative orbital computerized tomography scan

In the interim, the patient was lost to follow-up, but returned 20 days after, when it was noted that there was recurrence of total hyphema, with staining of the corneal graft, shown in Figure 3 (Fig. 3). Visual acuity returned to light perception, extraocular muscle movements decreased to 2/4. As seen in Figure 4 (Fig. 4), there was extensive thinning of the sclera, bloody chemosis, and proptosis. Computerized tomography scan of the orbit (Figure 5 (Fig. 5)) was done and showed the proptosed right globe, with peribulbar radiopaque densities, possibly new/fresh hemorrhage. Ocular ultrasound showed development of vitreous hemorrhage, with peripheral retinal detachment but a normal axial length.

Therefore, the patient sustained delayed rebleeding intraocularly as well as developed new onset bleeding in a new location postoperatively. Rebleed is a common occurrence postoperatively, at around 5–13%. Development of a new site of bleeding, however, is much rarer, and should alert physicians of an underlying bleeding disorder [8]. At this point, further workup and investigation of the patient was done.

**Figure 6:**
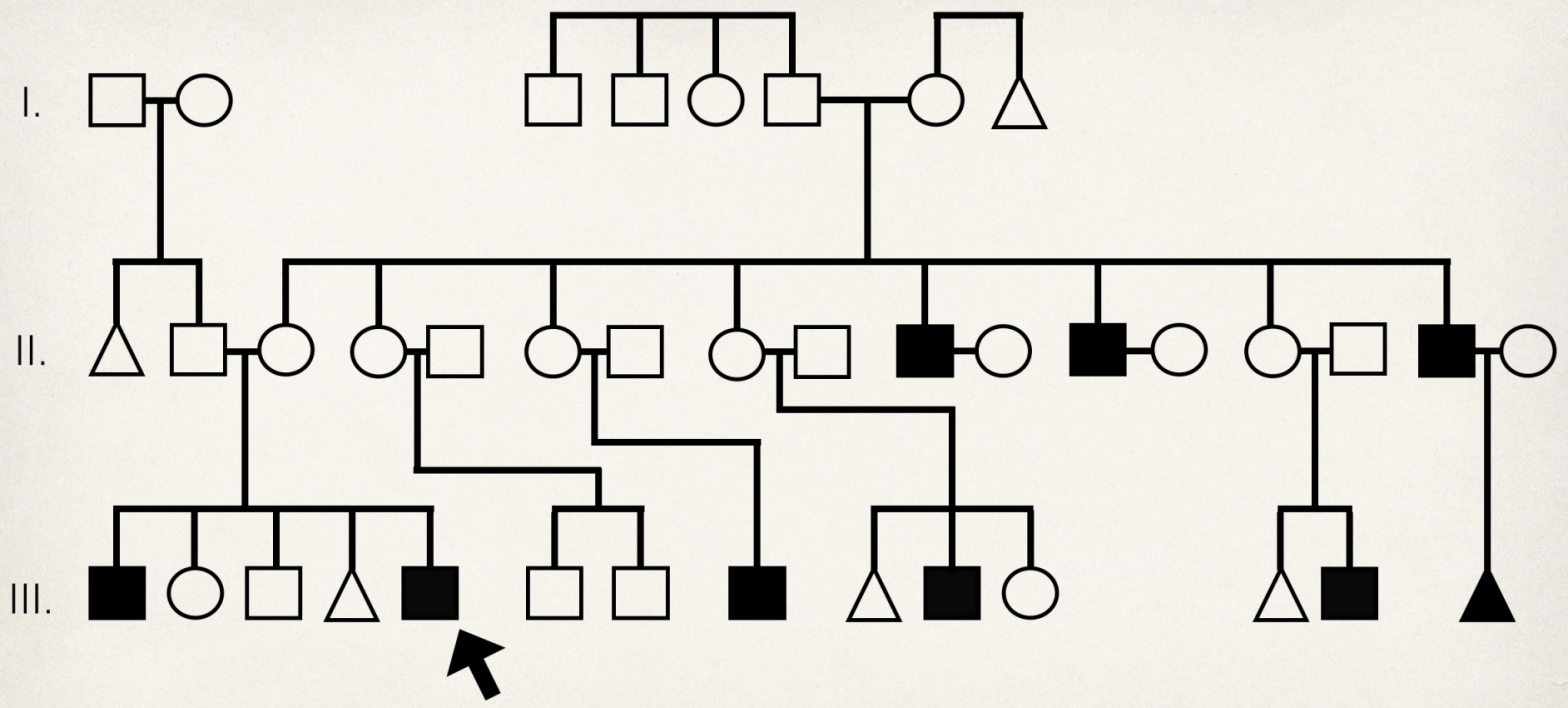
Family genogram showing X-linked inheritance pattern of bleeding disorder

On further probing with the patient’s parents, it was found that the patient actually had a history of easy bruisability from blunt trauma, but no consults had ever been done. Also, while having undergone a tooth extraction in 2006, the patient had sustained blood loss requiring transfusion. His relatives also allegedly had history of easy bruisability. A genogram was constructed, presented in Figure 6 (Fig. 6), which showed an X-linked inheritance pattern of bleeding in the patient’s family, wherein:

1. bleeding history affected only male members;
2. bleeding was inherited maternally;
3. no male-to-male transmission occurred;
4. affected males would transmit to all daughters, as in the 3^rd^ male sibling of the patient’s mother.

This history pointed to a possible inherited bleeding disorder, and workup was done. Tests showed increased time for activated partial thromboplastin time, which implies that a possible intrinsic or common pathway defect was present. Specific factor assays were then ordered to confirm diagnosis. The patient was found to be factor VIII deficient, which is ultimately confirmatory for hemophilia A and matches with the patient’s additional history. At this point, no further surgical intervention was done. The patient was transferred to Pediatrics for further management and transfusion of factor correction.

## Discussion

For bleeding disorders, laboratory testing of hemostatic function is warranted [1]. The diagnosis of hemophilia had been missed, since the standard workup for clearance in the hospital only included complete blood count and urinalysis, which would not detect the presence of bleeding disorder. Therefore, when there is high clinical suspicion, additional tests should always be done prior to any surgical intervention.

With regard to the surgery, various recommendations for surgical evacuation of hyphemas have been published [9]. In general, surgery is indicated for

1. risk of optic atrophy from increased intraocular pressure (evacuate if IOP>60 mmHg in 2 days or >35 mmHg in 7 days);
2. risk of corneal staining (evacuate if IOP>25 mmHg in 5 days or early evidence of staining);
3. risk of synechia formation (evacuate if >50% in 8 days or total hyphema for 5 days or more);
4. amblyopia prevention in children.

In this case, the patient had total hyphema and early evidence of staining at 10 days post-trauma, hence surgery was done. Surgery in hemophiliac patients is still possible, despite higher risk of bleeding. This risk is actually paradoxically attributed to the fact that the presence of a mild to moderate bleeding disorder often goes undiagnosed prior to the surgery, with the bleeding only discovered intraoperatively or postoperatively [6].

In any case, treatment of hemophilia starts with early recognition and prophylaxis prior to any anticipated surgery, as should have been the case in this patient. The primary goal of treatment in hemophilia is to increase the plasma levels of the deficient factor, at an at least 30–50% increase in moderate cases [1], [2]. This could be achieved either through releasing all endogenously available factor levels through intranasal desmopressin acetate; or, if severely limited, transfusion of recombinant factor or fresh plasma can be done.

With this advent of factor transfusion, ocular surgery had lower risks of developing complications intra- or postoperatively [10]. However, no consensus exists in various studies as to what levels of factor VIII or IX are acceptable preoperatively, as long as prophylactic treatment is started [11]. In ocular surgery, it is recommended that factor levels be raised to 70–80%, with a minimum of 50% in emergency cases [2].

Few reports have been published showing different outcomes of ocular surgeries in hemophiliacs. For elective surgeries for example, Fabian et al. [12] reported a case series of seven known hemophiliac patients who all underwent elective phacoemulsification without pre-treatment with factor correction. Surgery was uneventful for all cases; no minor or major bleeding episodes noted intraoperatively. Patients were followed-up for 1 week and no complications were noted. For emergency cases, a 2001 case series by Jijina et al. [13] highlighted 5 cases of hemophiliacs that underwent ocular surgery. Two cases were of boys less than 10 years old who both sustained minor blunt ocular trauma and developed traumatic hyphemas. In one case, the patient was a known severe hemophiliac prior to the trauma, and underwent factor transfusion correction prior to surgery. Recovery was reported to be uneventful. The other patient, however, had not been a known hemophiliac prior to surgery. Due to rebleeding postoperatively, the patient was investigated and found to be of moderate hemophilia type B. Correction was done prior to a second surgery for the rebleed, but then the patient developed vitreous hemorrhage postoperatively. At this point, the patient was treated medically with further transfusions, and the hemorrhage resolved and vision recovered.

These show that risk or complications in hemophiliac surgery can occur due to the fact that hemophilia is usually undiagnosed prior to the surgery, especially in cases of moderate hemophilia. These cases also illustrate that whether patients are treated preoperatively or not, outcomes still differ per patient, also regardless of the severity of the deficiency. Hence, meticulous postoperative monitoring and follow-up should always be done. Each case and the decision whether to operate or not should also always be individualized. Factor correction can help prevent complications, but the need and response depend on a case-to-case basis.

## Conclusion

In the end, once hemophilia A was confirmed on our particular patient, no further surgical intervention was done, prognosis was advised to the patient and parents, and the patient was transferred to Pediatrics for further medical treatment and transfusion. The patient was discharged from their service with uneventful recovery. On patient follow-up 6 months post-trauma, no new bleeding events were noted in the interim. The patient’s eye had already developed stained cornea and poor vision. However, no new signs of fresh bleeding were noted and intraocular pressure remained normal, no proptosis or chemosis were noted.

Inherited bleeding disorders are potentially life-threatening conditions that are underdiagnosed and can pose problems for the ophthalmologist. Seemingly frequent ocular symptoms could actually be a rare manifestation of hemophilia. Hence ophthalmologists should be more vigilant rather than dismissing these easily. Given the limited number of published reports on ocular hemorrhage in hemophiliacs, management of such patients should always be on an individualized basis, with emphasis on closer monitoring and follow-up of such patients.

## Notes

### Competing interests

The authors declare that they have no competing interests.

### Informed consent

Informed consent was taken prior to this study, with approval from the hospital institutional review board.
